# *Drosophila melanogaster* as a prospective model organism for monkeypox virus research

**DOI:** 10.3389/fmicb.2025.1696236

**Published:** 2025-12-16

**Authors:** Mukarram Mudjahid, Andri Frediansyah, Youdiil Ophinni, Firzan Nainu

**Affiliations:** 1 Department of Pharmacy, Faculty of Pharmacy, Hasanuddin University, Makassar, Indonesia; 2 Unhas Fly Research Group, Faculty of Pharmacy, Hasanuddin University, Makassar, Indonesia; 3 Research Center for Food Technology and Processing, National Research and Innovation Agency (BRIN), Yogyakarta, Indonesia; 4 The Hakubi Center for Advanced Research, Kyoto University, Yoshida-honmachi, Sakyo-ku, Kyoto, Japan; 5 Center for Southeast Asian Studies (CSEAS), Kyoto University, Sakyo-ku, Kyoto, Japan; 6 Immunology Frontier Research Center (IFReC), Osaka University, Suita, Osaka, Japan; 7 Center for Infectious Diseases (CID), Kobe University, Chuo-ku, Kobe, Japan

**Keywords:** fruit fly, host-pathogen interaction, disease model, monkeypox virus, antiviral drug screening

## Introduction

Since May 2022, the World Health Organization (WHO) has reported an unprecedented monkeypox virus (MPXV) outbreak affecting multiple non-endemic regions (Zumla et al., 2022). This outbreak is marked by atypical transmission dynamics, allowing sustained spread across areas historically unaffected by the virus. As of June 2025, 158,425 confirmed cases had been reported across 138 countries (WHO, 2025). Phylogenetic analyses identify two clades: Clade I (Central African) and Clade II (West African), differing in geography, clinical severity, and fatality rates (Hermez et al., 2024). Clade I, largely confined to Central and Eastern Africa, causes more severe disease, including encephalitis and sepsis, with fatality rates of 5%−10%. In contrast, Clade II, responsible for most recent cases, has lower virulence (<3% fatality rate) but higher human-to-human transmissibility, driving rapid global spread (Satheshkumar et al., 2025; Srivastava et al., 2025). Since January 2024, a total of 42,211 Clade IB infections have been reported, with the majority occurring in the Democratic Republic of the Congo (28,874 cases), followed by Uganda (7,874 cases) and Burundi (4,430 cases) (WHO, 2025).

The MPXV outbreak highlights the complex interplay between viral evolution (Zhang et al., 2024), ecological factors (Ogunleye et al., 2023), and host immune responses in driving disease emergence (Yi et al., 2024). Despite its genetic similarity to the variola virus (Chen et al., 2025), the causative agent of smallpox, no specific or effective antiviral treatment is currently available for MPXV (Ghosh et al., 2023). These gaps underscore the urgent need for further research into the virus's biology, pathogenesis, and control strategies. Amid growing concerns about the potential for another pandemic and the limited availability of therapeutic options, a comprehensive understanding of MPXV is essential. Experimental models that facilitate the efficient investigation of infection mechanisms and host responses are critically needed, not only to support the development of more effective therapeutic and preventive strategies but also to identify and validate targets for antiviral drug screening against MPXV.

## Biology, transmission, and challenges in modeling MPXV infection

MPXV, a member of the Poxviridae family, possesses a large double-stranded DNA genome of approximately 190 kilobases, encoding nearly 190 proteins (Shchelkunov et al., 2002). Mature virions are ovoid to brick-shaped, featuring surface tubules and a characteristic dumbbell-shaped nucleoprotein core that encloses the viral genome (Shchelkunov et al., 2001). MPXV particles typically measure around 200 nm in diameter and 300 nm in length (Wenner et al., 1968). As an enveloped virus, MPXV exhibits a complex life cycle involving both intracellular and extracellular forms (Lum et al., 2022). Like other poxviruses, replication occurs entirely in the cytoplasm of host cells, where viral factories are formed to support genome replication and virion assembly (Yi et al., 2024).

The transmission of the virus primarily occurs through direct contact with infected animals, bodily fluids, or lesion material (Chen et al., 2025). Close contact facilitates the spread of MPXV in both endemic and non-endemic regions. MPXV enters the host through oral, respiratory, or cutaneous routes, infecting mucosal surfaces or penetrating compromised skin. Once inside, the virus replicates within keratinocytes, fibroblasts, and endothelial cells (Di Giulio and Eckburg, 2004; Damon, 2011). During the initial stage of MPXV infection, viral proteins primarily mediate DNA replication and interact with host targets, leading to antiviral suppression and cell cycle arrest. These proteins are classified into three groups: (1) entry proteins (*M1R, E8L, H3L*) that enable host cell attachment and membrane fusion; (2) exit proteins (*A38R, C23R, C18L*) that facilitate virion release; and (3) immunomodulatory proteins (*J2L, F3L, A41L, P1L*) that alter host cell processes and immune responses to support viral persistence (Wang et al., 2024; Deng et al., 2025). In the late stage of MPXV infection, these proteins are crucial for the assembly of new viral particles. MPXV can spread to regional lymph nodes and subsequently to secondary organs such as the tonsils, spleen, and liver. Viral replication in these tissues triggers secondary viremia, leading to systemic dissemination to distant organs—including the lungs, kidneys, intestines, and skin. This widespread infection results in the characteristic clinical manifestations of Mpox (Di Giulio and Eckburg, 2004; Damon, 2011). Notably, the pathogenicity of human disease is associated with differences between viral strains (Likos et al., 2005).

Research on MPXV and the development of targeted antivirals remain challenging due to limited understanding of its pathogenesis, viral evolution, and immune evasion (Fang et al., 2024). Unlike variola virus, which was extensively studied during the smallpox eradication campaign, MPXV remains underexplored, leaving key gaps in its biological characterization. Progress is further constrained by reliance on traditional animal models such as non-human primates (NHPs) and rodents (Wei et al., 2023). Although these models have advanced knowledge of MPXV pathogenesis, they present major ethical, logistical, and financial limitations (Pastorino et al., 2024). NHP research requires stringent ethical oversight and high-containment BSL-3/4 facilities, making it costly and difficult to scale (Prescott et al., 2021). Rodent models, while more accessible, also demand significant maintenance resources (Hylander et al., 2022). These challenges highlight the urgent need for alternative, cost-effective, and ethically sustainable model systems to accelerate MPXV research and therapeutic discovery.

## *Drosophila* as a model organism for studying MPXV virulence and screening of prospective antivirals

*Drosophila melanogaster* has become a well-established and versatile alternative model for studying viral infectious diseases. Its significance in biomedical research lies in the evolutionary conservation of innate immune signaling pathways and fundamental cellular processes that are commonly targeted by viruses. For instance, Toll-like receptor signaling, which mediates pathogen recognition and activation of antiviral responses, plays a critical role in both flies and humans (Valanne et al., 2011). Similarly, *D. melanogaster* employs RNA interference (RNAi) as a primary antiviral defense mechanism, an evolutionarily conserved process that also contributes to antiviral immunity in mammals (Ding and Voinnet, 2007). These conserved pathways enable the use of *D. melanogaster* as a surrogate system to explore host–virus interactions at the molecular and cellular levels, offering insights that are translatable to higher organisms.

In addition to its immunological relevance, *D. melanogaster* offers several practical and technical advantages that make it particularly suitable for virological and genetic studies. The species is small, inexpensive to maintain, and can be reared in large numbers under simple laboratory conditions using easily prepared media (Victor Atoki et al., 2025). A single female can lay up to 100 eggs per day and around 2,000 during her lifetime (Giansanti et al., 2025). It also has a rapid life cycle of approximately 10–12 days at 25 °C, allowing rapid generation turnover (Baenas and Wagner, 2019). These features make *D. melanogaster* ideal for large-scale and high-throughput experiments. In terms of ethical considerations, *D. melanogaster* research is subject to fewer regulatory restrictions compared to vertebrate or mammalian models. The National Center for the Replacement, Refinement and Reduction of Animals in Research (NC3Rs) is a UK-based organization promotes the 3Rs principles (Replacement, Reduction, and Refinement) to ensure ethical and humane research practices (NC3Rs, n.d.; European Union, 2010). In line with these principles, the NC3Rs encourages the use of invertebrate models such as *D. melanogaster*, nematodes, and social amoebae, which are generally not considered suffering, thereby reducing ethical concerns and simplifying institutional approval (NC3Rs).

From a genetic perspective, *D. melanogaster* represents one of the most genetically accessible model organisms, supported by a comprehensive suite of genetic tools that enable precise and targeted genome manipulation. This is further supported by the fact that *D. melanogaster* has fewer genes than humans (flies ~14,000 genes vs. humans ~20,000), implying less genetic redundancy and facilitating easier examination of gene function (Hughes et al., 2012). Recent advancements, particularly the implementation of CRISPR/Cas9, the GAL4/UAS system, and RNAi, have enabled precise gene manipulation in a tissue-specific manner (Hughes et al., 2012; Zirin et al., 2022; Meltzer et al., 2019; Mascolo et al., 2022; Qiao et al., 2018; Link et al., 2024; Querenet et al., 2015). These genes can be knocked out or modified to observe how changes in the expression of those genes can influence infection. This combination of biological relevance, cost-efficiency, ethical accessibility, and genetic tractability, makes *D. melanogaster* an important and practical model for investigating viral pathogenesis, host responses, and the molecular mechanisms underlying infection.

## Discussion

MPXV has been shown to induce substantial tissue damage and, in severe instances, lead to mortality in humans. However, the molecular basis of its pathogenicity remains poorly understood. We hypothesize that the pathogenicity of MPXV is primarily driven by viral–host protein interactions that modulate key physiological signaling pathways within the host. Such interactions may alter cellular homeostasis, immune responses, and metabolic regulation, ultimately contributing to disease manifestation. For example, the *P1L* gene in MPXV, which is homologous to the *N1L* gene in vaccinia virus (VACV), functions as an anti-apoptotic factor and a modulator of host defense. It has been shown to inhibit NF-κB and IRF3 signaling pathways, both of which play critical roles in orchestrating effective immune responses against viral infection (Deng et al., 2025).

**Figure 1:**
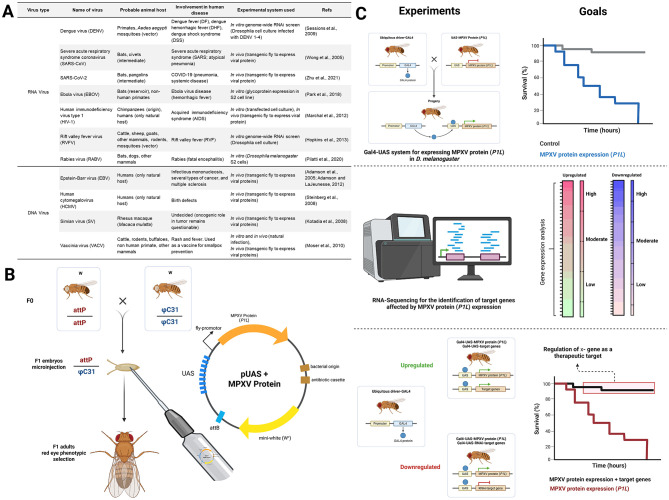
Overview of the prospective model organism for MPXV research. **(A)** Human viruses studied using *Drosophila melanogaster*. **(B)** Representative schematic illustrating UAS-mediated expression of the MPXV protein (example, *P1L*) in *D. melanogaster* transgenic lines. The MPXV *P1L* protein, functions as an anti-apoptotic and immune-modulatory factor by inhibiting NF-κB and IRF3 signaling pathways, which are essential for antiviral responses. **(C)** Basic design of experimental approaches utilizing *D. melanogaster* to identify potential therapeutic targets for MPXV. Created in BioRender. Nainu, F. (2025) https://BioRender.com/qj7pv6u.

To better elucidate the underlying mechanisms, researchers can use a comprehensive toolkit of transgenic *D. melanogaster* lines that enable the expression of viral proteins using the GAL4/UAS binary expression system (Link et al., 2024) (see Figure 1C). Although *D. melanogaster* is not a natural host for MPXV, previous studies have demonstrated that a variety of human viral infections, including both DNA and RNA viruses, can be effectively modeled in *D. melanogaster* (see Figure 1A) (Sessions et al., 2009; Wong et al., 2005; Zhu et al., 2021; Park et al., 2018; Marchal et al., 2012; Hopkins et al., 2013; Pilatti et al., 2020; Adamson et al., 2005; Adamson and LaJeunesse, 2012; Steinberg et al., 2008; Kotadia et al., 2008; Moser et al., 2010). Notably, several of these reported models have successfully expressed viral proteins in transgenic *D. melanogaster* using the GAL4–UAS system. The GAL4/UAS system allows controlled expression of individual viral genes in specific tissues, facilitating the investigation of their molecular and physiological effects without requiring live viral infection. By expressing MPXV candidate genes such as *P1L* in targeted *D. melanogaster* tissues, researchers can systematically assess their impact on host signaling pathways, thereby gaining insights into the molecular mechanisms underlying MPXV-induced pathogenesis and potentially identifying novel therapeutic targets. This approach is particularly advantageous for studying viruses like MPXV that possess restricted host ranges and limited infectivity.

The initial step involved designing transgenic fly lines that express specific viral proteins (see Figure 1B). Genes encoding these viral proteins were first codon-optimized for efficient translation in *D. melanogaster* and subsequently cloned into UAS expression vectors. The resulting constructs were integrated into the fly genome via integrase or transposon-mediated transformation to generate UAS–viral protein transgenic lines. These lines were then crossed with tissue-specific GAL4 driver strains to achieve precise spatial and temporal control of viral protein expression. This platform enables *in vivo* characterization of viral protein functions, host–protein interactions, and associated cytotoxic effects. The expressed viral proteins are further evaluated for toxicity phenotypes, which can be quantified through survival analysis to determine their physiological impact *in vivo*.

Global host gene responses to viral protein expression are then assessed using RNA sequencing to identify significantly dysregulated genes. Candidate genes identified through transcriptomic analysis are functionally validated using a rescue-based experimental design employing a dual Gal4/UAS system. This approach allows simultaneous manipulation either through overexpression or knockdown (RNAi) of target genes (Zhang et al., 2020), alongside MPXV protein expression to evaluate their protective or pathogenic roles. An improvement in survival following gene manipulation indicates a potential therapeutic role, whereas a further decrease in survival suggests that the gene may exacerbate MPXV-induced toxicity. This integrative approach may offer a robust and scalable platform for identifying critical host and viral factors interaction, thereby facilitating the rational design of targeted therapeutics against MPXV infections while enhancing our mechanistic understanding of viral pathogenesis.

Despite its numerous advantages, *D. melanogaster* has inherent limitations as a model for therapeutic target screening in MPXV research. According to the DRSC Integrative Ortholog Prediction Tool (DIOPT), which assesses genetic conservation between flies and humans, the number of genes associated with viral diseases is relatively limited. Specifically, 65 human genes are linked to viral diseases, but only 39 of these have identified orthologs in *D. melanogaster* (Hu et al., 2011). This indicates that only a limited subset of human genes has direct counterparts in flies, likely due to fundamental biological differences such as the absence of an adaptive immune system. Moreover, significant physiological differences, including the lack of certain mammalian organ systems and distinct tissue microenvironments, further constrain the ability to replicate disease phenotypes directly. These limitations highlight the need for subsequent validation in mammalian models and human cell–based systems to confirm the functional conservation of the observed mechanisms. This can be achieved through the analysis of gene and protein expression of human homologs under MPXV natural infection. Such validation would determine whether the molecular targets identified in *D. melanogaster* exhibit comparable expression patterns and functional roles in mammalian or human systems. By integrating genetic and virological perspectives through the use of the *D. melanogaster* model, this study emphasizes the value of interdisciplinary approaches in uncovering molecular mechanisms that bridge fundamental genetic insights with virological research.
